# Massive/massebildende duktuläre Reaktion (MDR) bei angioinvasivem hepatozellulärem Karzinom: Pathogenese und Differenzialdiagnosen eines hepatischen Pseudotumors

**DOI:** 10.1007/s00104-026-02530-0

**Published:** 2026-06-23

**Authors:** Anne Kristin Fischer, Reinhard Büttner, Thorsten Persigehl, Hans-Peter Fischer

**Affiliations:** 1 Institut für Pathologie, Universitätsklinik Köln, Kerpener Str. 62, 50937 Köln, Deutschland; 2 Institut für Diagnostische und Interventionelle Radiologie, Universitätsklinik Köln, Kerpener Str. 62, 50937 Köln, Deutschland; 3 Institut für Pathologie, Universitätsklinik Bonn, Venusberg Campus 1, 53127 Bonn, Deutschland; 4 Medizinisches Versorgungszentrum Pathologie und Zytologie Rhein-Sieg, Mendener Str. 12, 53830 Troisdorf, Deutschland

**Keywords:** Leberumbauprozess, Pseudotumoröse Leberläsion, Obstruktion des hepatischen Blutflusses, Gallengangstumoren, Leberbioptische Abgrenzung, Liver remodelling process, Pseudotumoral hepatic lesion, Obstruction of the hepatic circulation, Cholangiocarcinoma, Differentiation by liver biopsy

## Abstract

Die massive/massebildende duktuläre Reaktion (MDR) der Leber ist eine Form ausgedehnten parenchymatösen Remodellings unter der Bedingung fokaler relativer Ischämie. Diese pseudotumoröse Reaktion des Leberparenchyms wird durch eine lokal ausgedehnte Okklusion hepatovenöser, portovenöser und sinusoidaler Blutgefäße hervorgerufen. Oft ist eine angioinvasive Obliteration durch ein koinzidentes Malignom die Ursache. Aber auch ein Auftreten im nichtmalignen Kontext in einer Leberzirrhose oder nach ausgedehnten Lebernekrosen ist möglich. Nicht immer gelingt die sichere präinterventionelle Einordnung, sodass eine leberbioptische Abgrenzung notwendig ist.

## Abstract (en)

Massive/mass-forming ductular reaction (MDR) of the liver is a form of extensive parenchymatous remodelling under conditions of focal relative ischemia. This pseudotumoral reaction of the liver parenchyma is induced by locally extended occlusion of hepatic venous, portal venous and sinusoidal blood vessels. It is often caused by an angioinvasive obliteration from a coincidental malignant tumor but an occurrence in a benign context in a liver cirrhosis or after extensive liver necrosis is possible. The certain preinterventional classification is not always possible so that a differentiation with a liver biopsy is necessary.

## Anamnese, klinische Befunde, Therapie und Verlauf

**Abb. 1:**
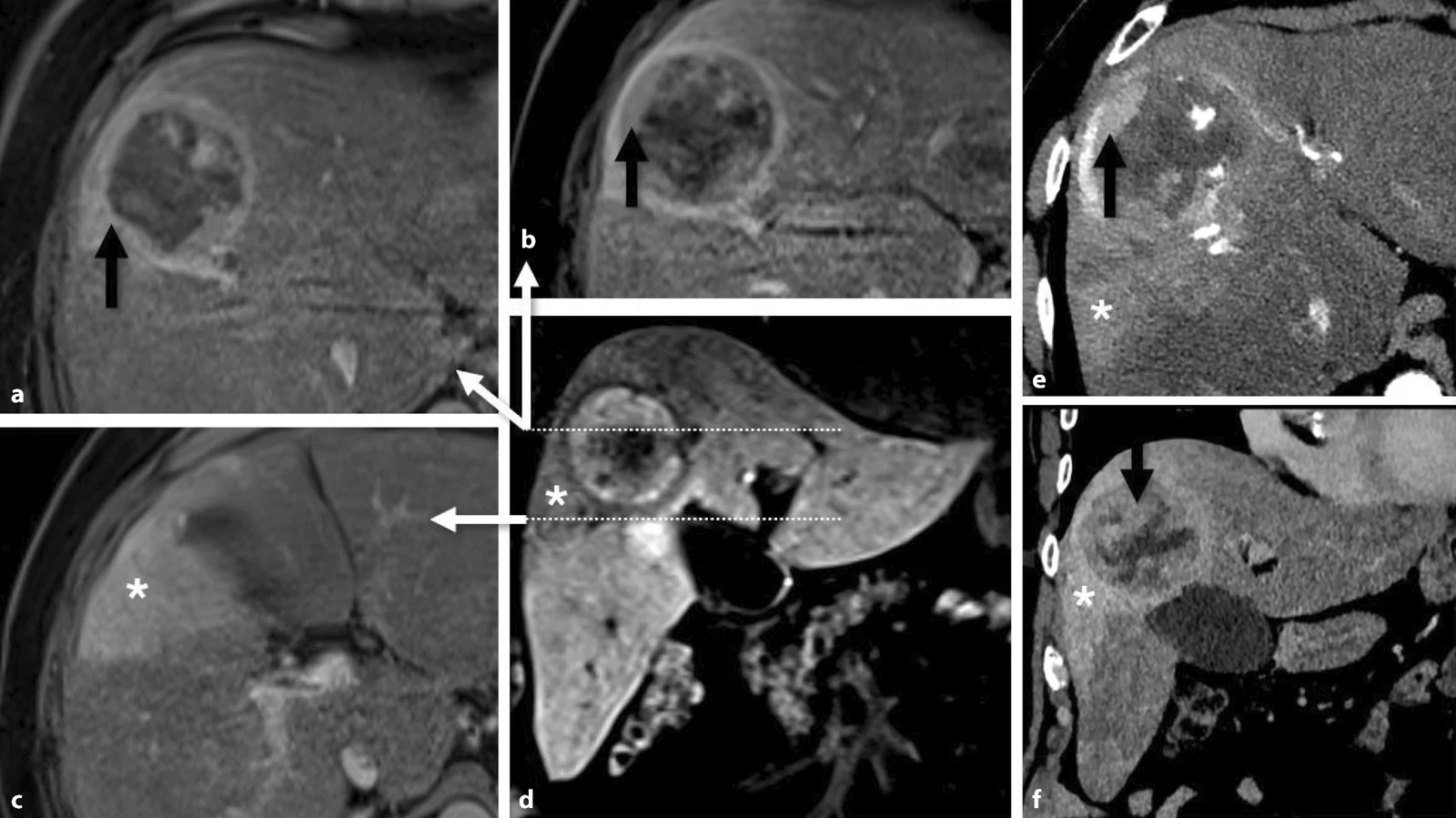
**a**–**d** Leber-MRT und **e**, **f** -CT. In der Leber-MRT unter Verwendung des hepatospezifischen Kontrastmittels (Gadoxetsäure; Primovist^TM^) zeigt sich intraläsional in der Leberraumforderung im Segment VIII (**a**, **b** obere Ebene, gestrichelt) in der **a** arteriellen Phase ein zirkuläres randständiges Kontrastmittel-Enhancement (*Pfeil*). Dieses weist **b** ab der portal-venösen Phase ein Wash-out-Phänomen auf (*Pfeil*) bei angrenzender schmaler Pseudokapsel sowie **d** mit einem in der hepatobiliären Phase detektierbaren Primovist^TM^-Uptake. Angrenzend an die Leberraumforderung (**c** untere Ebene, gestrichelt) zeigt sich peritumoral eine flächige arterielle bis portal-venöse Hyperperfusion mit **d** in der Spätphase nach 20 min vermindertem Primovist^TM^-Uptake (*Stern*). Im CT **e**, **f** zeigen sich analog zur MRT **e** intraläsional eine randständige arterielle Hyperperfusion (*Pfeil*) mit ab der portal-venösen bis zur späteren Phase **f** geringem Wash-out-Phänomen (*Pfeil*) sowie zentral einzelne arterielle Tumorgefäße mit zunehmend protrahiertem Kontrastmittel-Enhancement (**e**, **f**). Peritumoral stellt sich ebenfalls ab der arteriellen Phase eine regionale Hyperperfusion im Lebersegment VIII und V dar (*Stern*), die bis zur Spätphase nach 4 min persistiert

Bei einem 68-jährigen Mann fand sich bei bildgebender Verlaufskontrolle einer Fettleberzirrhose eine tumoröse Läsion in Segment VIII. In der externen Sonographie und in der Leber-Magnetresonanztomographie (MRT) mit hepatozellulärem Kontrastmittel (Primovist^TM^, Bayer Vital GmbH, Leverkusen, NRW, Deutschland) zeigte sich eine große Leberraumforderung, in der jeweiligen Untersuchung 8,2 × 8,1 cm bzw. 7,3 × 7,1 cm messend. Sonographisch lag eine arteriell flau und randständig kontrastmittelaufnehmende, spät-venös inhomogene, teils hyperechogene, zentral hypoechogene Leberläsion vor. Angrenzend fand sich ein bis nach kaudal ziehendes hyperechogenes Areal. Im Primovist^TM^-MRT wies die Raumforderung nativ eine inhomogene, aber glatt begrenzte Kapsel auf. Nach Kontrastmittelapplikation zeigte sich in der arteriellen Phase ein zirkuläres randständiges Enhancement, das ab der portal-venösen Phase ein Wash-out-Phänomen aufwies bei angrenzender schmaler Pseudokapsel mit wiederum in der hepatobiliären Phase nachweisbarem hyperintensem Primovist^TM^-Uptake. Die zentralen Areale waren überwiegend nicht perfundiert mit nur kleineren hyperperfundierten Regionen. Angrenzend an die Leberraumforderung zeigte sich peritumoral eine flächige arterielle bis portal-venöse Hyperperfusion mit vermindertem Primovist^TM^-Uptake in der Spätphase nach 20 min. Extern wurde die Verdachtsdiagnose auf ein teilthrombosiertes Hämangiom geäußert, differenzialdiagnostisch auf ein hepatozelluläres Karzinom (HCC). Im lokalen Lebertumorboard wurde die Diagnose auf ein atypisches HCC mit peritumoralem Corona-Zeichen als Risiko für eine Mikrogefäßinfiltration [1] mit erhöhtem Rezidivrisiko gestellt und eine Leberresektion empfohlen. In der Staging-Computertomographie (CT) zeigten sich analog zur MRT intraläsional eine randständige arterielle Hyperperfusion mit portal-venös geringem Wash-out-Phänomen sowie zentral einzelne arterielle Tumorgefäße mit zunehmend protrahiertem, zentralem Kontrastmittel-Enhancement. Peritumoral stellte sich ebenfalls eine regionale Hyperperfusion im Lebersegment VIII und V dar, die bis zur Spätphase nach 4 min persistierte (Abb. 1).

Im leberspezifischen Labor fand sich eine milde Erhöhung der Transaminasen (AST/GOT 55,3↑ [R < 50 U/l]; ALT/GPT 83,6↑ [R < 50 U/l] und GGT 125↑ [R > 60 U/l]), bei im Übrigen weitgehend normwertigen Parametern.

Es erfolgte eine offene typische zentrale Lebersegmentresektion.

## Histopathologische Diagnostik

### Konventionelle histologische und immunhistochemische Analysen

Repräsentatives Tumor- und Umgebungsgewebe wurde histochemischen Färbungen unterzogen (Hämatoxylin-Eosin [HE], Period-Schiff-Säure [PAS], Diastase-Period-Schiff-Säure [D-PAS], Elastica van Gieson [EvG], Gordon/Gomori, Sirius und Berliner-Blau-Reaktion [Eisenablagerungen]). Die anschließende immunhistochemische Charakterisierung erfolgte mit Antikörpern gegen CK7 (Klon OV-TL12/30), CK19 (Klon RCK108), p53 (Klon DO-7) (alle Dako/CE), CD56 (Klon 123C3; Thermo/RUO), Ki-67 (Klon MIB1; ZetaCorp). Die Färbungen wurden halbautomatisch mit dem BenchMark Immunostainer (Ventana, Tucson, AZ, USA) durchgeführt.

## Pathologischer Befund

**Abb. 2:**
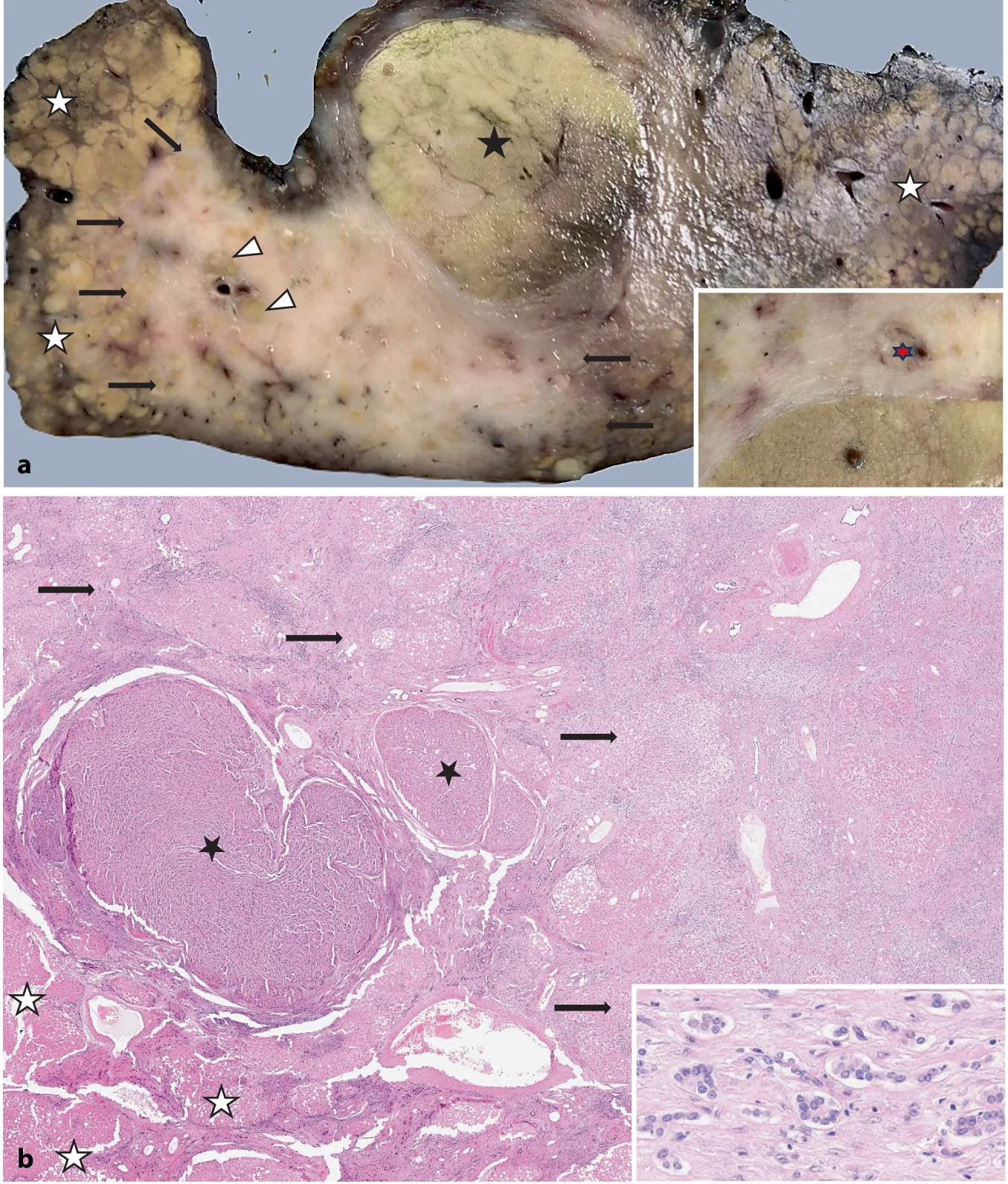
**a** 5,0 cm messende massive/massebildende duktuläre Reaktion (MDR, *schwarze Pfeile*) auf dem Boden ausgedehnter Portalvenen- und Zentralvenenastverschlüsse (Inset; *roter Stern*) bei benachbartem 6,5 cm messendem, konventionellem hepatozellulärem Karzinom (*schwarzer Stern*) und fortgeschrittener Leberzirrhose (links unten, *weiße Sterne*). In der MDR sind fokal kleine residuelle Leberparenchymknoten neben einem nicht obliterierten Blutgefäß enthalten (*weiße Pfeile*). **b** HE-Übersicht der MDR (rechte und obere Bildhälfte; *Pfeile*) auf dem Boden ausgedehnter Pfortaderastthrombosen (links mittig und unten) durch das HCC (*schwarze Sterne*). Inset: Duktuläre Proliferate mit schmalem Zytoplasma, leicht formvarianten Zellkernen mit zartem Chromatin und einzelnen winzigen Nukleolen. Im lockeren Stroma finden sich eingestreute Entzündungszellen

*Makroskopisch *fand sich in einer mittel- bis großknotigen Leberzirrhose ein ca. 10,0 cm messendes, aus 2 ganz unterschiedlichen Anteilen bestehendes tumoröses Areal:

- ein 6,5 cm messender zart gekapselter Lebertumor mit grünlicher oder blass-ockerfarbener Schnittfläche mit dem Aspekt eines HCC,
- damit breit verhaftet ein 5,0 cm messender, unscharf begrenzter, weiß-grauer derber Herd mit Reminiszenz an ein Cholangiokarzinom (Abb. 2a).

Letzteres Gewebeareal enthielt einzelne obliterierte größere Blutgefäße (Abb. 2a, Inset). Es umschloss darüber hinaus wenige grünliche Parenchyminseln.

**Abb. 3:**
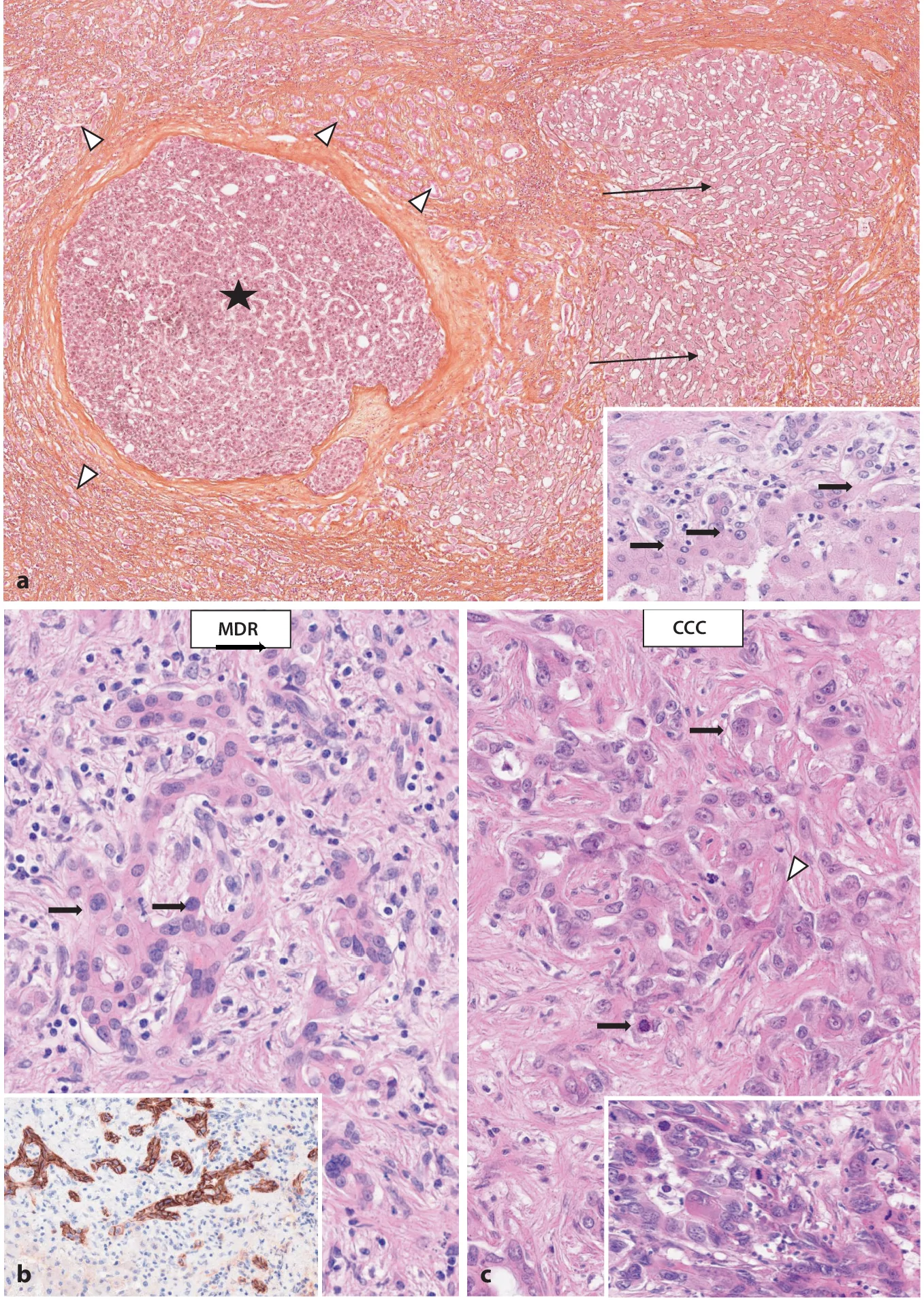
**a** MDR (*weiße Pfeile*) auf dem Boden einer Pfortaderastthrombose durch das HCC (*Stern*) in höherer Vergrößerung in der Gomori-Versilberungsreaktion. Atrophe Leberparenchymresiduen (*schwarze dünne Pfeile*) in Nachbarschaft des tumorösen Pfordaderastverschlusses sind verblieben und werden allmählich durch die duktuläre Reaktion ersetzt. Inset: Reaktive duktuläre Aussprossungen, sog. „finger tip-like endings“ in unmittelbarem Kontakt zu Hepatozyten der portoparenchymatösen Übergangszone im Bereich Hering-Kanäle (*Pfeile*). **b**, **c** Starke morphologische Ähnlichkeiten zwischen MDR (Abb. 3b) und einem gut differenzierten cholangiozellulären Karzinom (anderer Casus zum Vergleich) (**c**). Beide Läsionen bestehen aus schmalsträngigen duktulären Formationen, bei der MDR mit leichter Kernformvarianz, im Falle des CCC mit diskreten echten Atypien mit prominenten Nukleolen (*schwarze Pfeile*). Das Stroma des CCC erscheint faserreicher, die Tumordrüsen bilden lanzettförmige Enden aus (*weißer Pfeil*). Sowohl die MDR (*Inset links unten*) als auch das CCC exprimieren immunhistochemisch CD56 als Ausdruck einer gewissen Stammzellnähe, die sich auch in der morphologischen Unreife der reaktiven Duktulusproliferate widerspiegelt. An anderer Stelle weist das CCC ausgeprägtere Atypien auf und ist somit sicher als maligner Tumor einzuordnen (*Inset rechts unten*)

*Histologisch *bestand das große weißliche, tumorös imponierende Areal neben dem HCC aus duktulären Proliferaten, eingebettet in einer lockeren, fein-fibrillären Matrix mit eingestreuten Entzündungszellen. Darin gelegene größere Portal- und Lebervenenäste waren durch Proliferate des benachbarten HCC verschlossen (Abb. 2b). In diesen minderperfundierten Zonen ersetzten die Duktulusproliferate zugrunde gegangenes Leberparenchym. Nur wenige atrophe Parenchyminseln waren in Nachbarschaft spärlicher noch offener Blutgefäße erhalten (Abb. 2b und 3a). Die duktulären Strukturen zeigten eine gewisse morphologische Unreife. Sie bildeten verzweigte, schmale, meist lichtungslose Stränge mit fingerförmig abgerundeten Enden. Die rundovalen, leicht formvarianten Zellkerne enthielten ein fein-granuläres Kernchromatin mit gelegentlichen winzigen Nukleolen (Abb. 2b, Inset, Abb. 3a, Inset, Abb. 3b). Sie unterschieden sich somit im Reifegrad von echten Lichtung-führenden interlobulären Gallengängen der Portalfelder. Im periportoseptalen Grenzbereich zum Leberparenchym waren fingerförmige Aussprossungen neu gebildeter, biliär differenzierter Strukturen auf Höhe der Hering-Kanäle in unmittelbarem Kontakt zu Hepatozyten zu beobachten (Abb. 3a, Inset, Abb. 3b). Entsprechend ihrer stammzellnahen Differenzierung exprimierten die duktulären Proliferate durchgehend CD56 („neural cell adhesion molecule“ [NCAM]) (Abb. 3b, Inset), des Weiteren als Zeichen biliärer Differenzierung die Zytokeratinpolypeptide CK7 und CK19. Die Proliferationsaktivität war mit unter 3 % minimal; p53 zeigte ein Wildtyp-Expressionsmuster. In Abb. 3b, c wird die MDR (Abb. 3b) einem hoch differenzierten cholangiozellulären Karzinom mit dichterem desmoplastischem Stroma (Abb. 3c) gegenübergestellt (anderer Casus). Bei Verdacht auf ein hochdifferenziertes Karzinom ist die Suche nach atypiereicheren Arealen wichtig, wie der geringer differenzierte Tumorabschnitt im Inset in Abb. 3c zeigt, der die Malignität eindeutig belegt.

## Diagnose

Zentrales Leberresektat mit einem bis 6,5 cm messenden, mäßiggradig differenzierten, klassischen hepatozellulären Karzinom (HCC; G2) mit Hämangioinvasion in peritumorale Portalvenen- und Zentralvenenäste. Keine Lymphangio- oder Perineuralscheideninfiltration. Keine Infiltration der Leberkapsel. Resektion lokal in sano.

Molekularpathologisch Nachweis einer aktivierenden *CTNNB1*-Mutation (EX3: c.98 C > G; p.Ser33Cys; VAF 35,8) [13] im HCC. Kein Nachweis einer *hTERT*-Promotor-Mutation.

TNM-Klassifikation: pT2 (m), pNx, L0, V1, Pn0, R0.

5,0 cm messende, breit mit dem HCC verhaftete massive/massebildende duktuläre Reaktion (MDR) auf dem Boden hämangioinvasiver Portalvenen- und Zentralvenenastverschlüsse durch das HCC.

Vorbestehende fortgeschrittene stationäre mittel- bis großknotige Fettleberzirrhose.

## Verlauf

Der Patient konnte nach postoperativem Aufenthalt auf der Intensivstation nach Hause entlassen werden. In regelmäßigen Kontrolluntersuchungen ergab sich ein halbes Jahr später kein Hinweis auf ein Rezidiv des HCC.

## Hintergrund

Die massive/massebildende duktuläre Reaktion (MDR) [3] der Leber ist eine kürzlich beschriebene Form ausgedehnten parenchymatösen Remodellings unter der Bedingung fokaler relativer Ischämie. Diese pseudotumoröse Reaktion des Leberparenchyms (≥ 1,0 cm) wird durch eine lokal ausgedehnte Okklusion hepatovenöser, portovenöser und sinusoidaler Blutgefäße hervorgerufen. Oft ist eine angioinvasive Obliteration durch ein koinzidentes Malignom die Ursache, so bei hepatozellulärem oder cholangiozellulärem Karzinom (CCC), bei malignen Gefäßtumoren wie dem epitheloiden Hämangioendotheliom (HEH) oder dem hepatischen Angiosarkom (HAS) oder bei hepatisch angioinvasiven Karzinommetastasen [16]. Aber auch ein Auftreten im nichtmalignen Kontext in einer Leberzirrhose oder nach ausgedehnten Lebernekrosen ist möglich [4, 16]. MDR sind nicht nur bei koinzidenten malignen Lebererkrankungen eine Herausforderung für die Radiologie, die Chirurgie und die Pathologie. Nicht immer gelingt die sichere präinterventionelle Einordnung. Die leberbioptische Abgrenzung insbesondere gegenüber einem Cholangiokarzinom vom „small duct type“ oder „malformation type“, schließlich einem kombinierten hepatozellulären und cholangiozellulären Karzinom [4–6] ist besonders herausfordernd.

Duktuläre Reaktionen (DR) und nodulärer Parenchymumbau sind bekannte Folge einer chronisch gestörten Leberperfusion [7]. DR wurden histopathologisch erstmalig von Hans Popper et al. im Jahr 1957 definiert [8]. Es folgten zahlreiche Studien im Hinblick auf auslösende Leberschädigungen und die möglichen Ursprungszellen. Zu nennen sind hier insbesondere die Konzepte von Desmet, Roskams und Turányi et al. [9–11]. Die bis heute akzeptierte Definition von Roskams et al. aus dem Jahr 2004 beschreibt die DR als „eine Reaktion duktulären Phänotyps, möglicherweise aber nicht zwingenderweise duktulären Ursprungs, bei akuter oder chronischer Lebererkrankung“ [10]. Desmet untergliederte die DR [10] in 4 Typen, abhängig vom morphologischen Erscheinungsbild, der Komplexität, der Lokalisation, der Ausdehnung der Reaktion und der Art der Hintergrunderkrankung (Typ I, IIa, IIb, III), wobei der Typ III die ausgedehnteste, panazinäre Form der DR beschreibt [10]. Als mögliche Ursprungszellen werden

- präexistente Cholangiozyten,
- lokale oder zirkulierende Progenitorzellen (möglicherweise aus dem Knochenmark) oder
- biliäre Metaplasien von Hepatozyten

diskutiert [9–14]. DR können als Folge von Leberparenchymschäden unterschiedlicher Ätiologie auftreten. Dem Typ I liegt zumeist ein stenosierender Gallenwegsprozess, den Typen II und III jedoch eine Parenchymalteration mit gestörter Leberperfusion zugrunde. Histopathologisch ist eine gewisse Differenzierung der DR in morphologische Subtypen in Abhängigkeit von der Hintergrundschädigung möglich, wie von Desmet und Turányi et al. ausgeführt wurde [10, 12].

Mögliche Ursachen für eine DR sind:

- eine extrahepatische obstruktive Gallenwegserkrankung (oft sehr gut differenzierte DR, ähnlich normalen Gallengängen und in Kontakt mit diesen),
- Zirrhosen unterschiedlicher Ätiologie,
- ausgedehnte Lebernekrosen gefolgt von ausgeprägter Parenchymzellregeneration (differenzierende DR, kaum erkennbare Lumina bei Zirrhose, oft hepatobiliäre Metaplasien),
- chronische portale/periportale Leberentzündungen einschließlich intrahepatischer Gallenwegserkrankungen,
- intrahepatische Perfusionsstörungen,
- der Sonderfall einer fokal nodulären Hyperplasie (FNH) (undifferenzierte DR, flache Epithelien, kaum erkennbare Lumina).

**Abb. 4:**
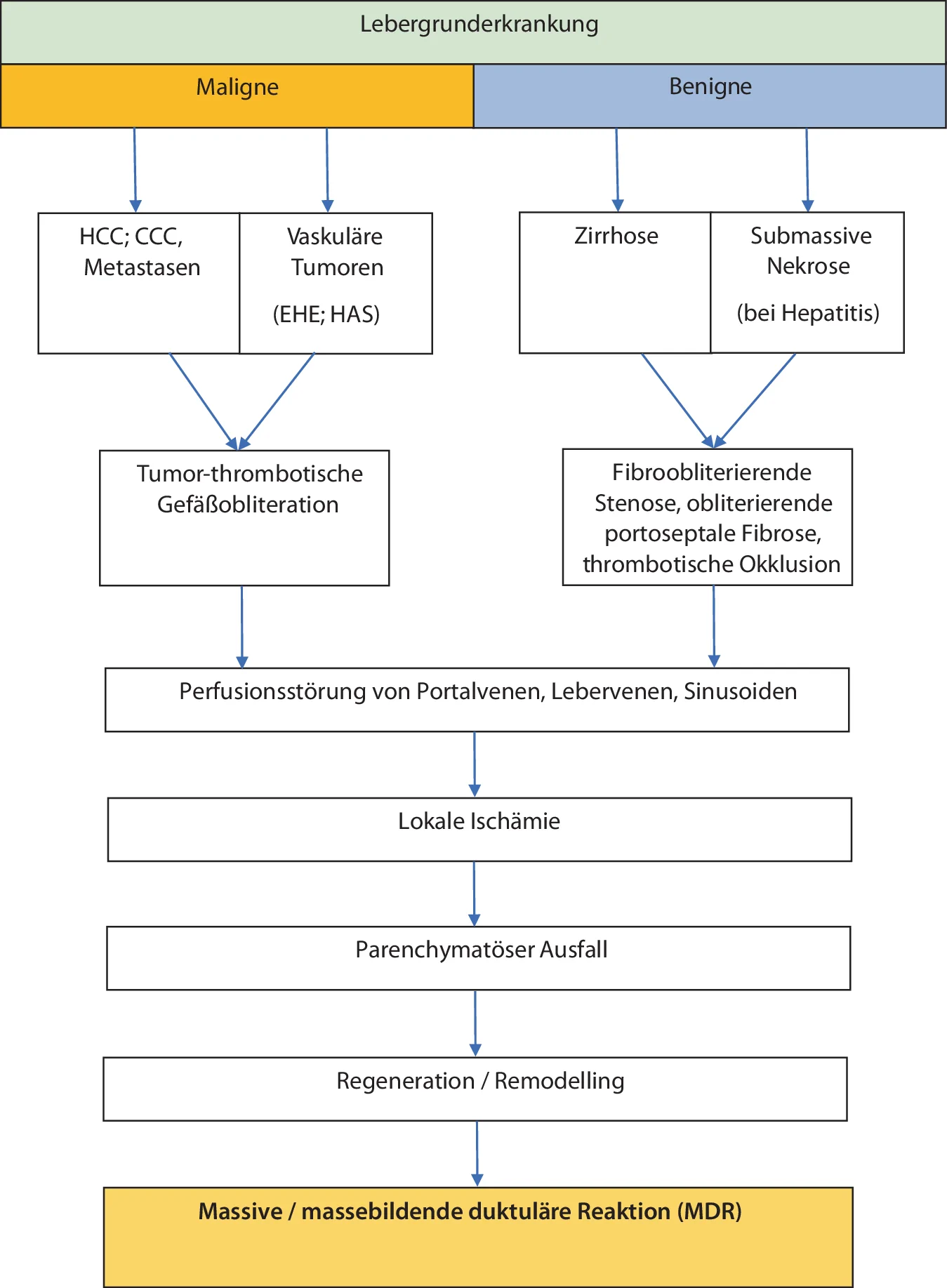
Entwicklung der MDR auf dem Boden einer lokalen relativen Ischämie durch Portal- und Lebervenenastverschlüsse sowie fibroobliterierende sinusoidale Obstruktion im Rahmen bislang definierter auslösender maligner und benigner Grunderkrankungen

Wir möchten anhand dieser Kasuistik die von uns kürzlich neu beschriebene massive/massebildende duktuläre Reaktion („massive/mass-forming ductular reaction“ [MDR]) [3] als eine seltene, klinisch relevante pseudotumoröse, pluriazinäre Gallengangsreaktion vorstellen. Die MDR führt nicht selten zu einer klinischen und histopathologischen Fehlinterpretation eines malignen Gallengangstumors. Sie erscheint uns deswegen in der radiologisch-chirurgischen Differenzialdiagnostik unklarer Leberknoten wichtig. Das angefügte Flussdiagramm (Abb. 4) soll die Entstehung der MDR im Kontext möglicher Lebergrunderkrankungen mit Perfusionsstörung verdeutlichen.

## Diskussion

In dem hier vorgestellten weiteren erstpublizierten Fall zeigen wir eine 5,0 cm messende MDR auf dem Boden einer lokalen relativen Ischämie, bedingt durch eine massive tumoröse Portal- und Lebervenenobliteration bei koinzidentem hepatozellulärem Karzinom in einer Zirrhose auf dem Boden einer früheren Fettleberhepatitis. Diese ungewöhnliche pseudotumoröse Läsion erschwert, – wie auch im gegebenen Fall – die präoperative bildgebende Einordnung. Sie stellt auch Pathologen/Pathologinnen vor erhebliche differenzialdiagnostische Probleme. Im Vordergrund steht die Abgrenzung gegenüber dem Cholangiokarzinom vom „small duct type“ und – wie hier dargestellt – gegenüber einem kombinierten hepatozellulären und cholangiozellulären Karzinom.

Grundlage der von uns erstmals beschriebenen MDR ist eine örtliche Minderperfusion des Lebergewebes durch Obliteration versorgender Portalvenen und/oder drainierender Lebervenen oder unzureichende sinusoidale Perfusion. Wir beobachteten die MDR

- im Rahmen einer subakuten Lebernekrose bei schwerer Early-onset-Autoimmunhepatitis,
- in Assoziation mit (in)kompletten Zirrhosen,
- bei malignen epithelialen Tumoren (HCC, CCC) und malignen vaskulären Tumoren (hepatisches Angiosarkom [HAS], epitheloides Hämangioendotheliom [EHE]) [3].

Zwischenzeitlich beschrieben Joldoshova et al. [16] 4 ähnliche Fälle biliärer Pseudotumoren, assoziiert mit Atrophie bei HCV- und Alkohol-assoziierter Leberzirrhose und Kolonkarzinommetastasen. Knotige duktuläre Proliferate, die Ähnlichkeit mit einem gut differenzierten CCC zeigten, ersetzten in den von ihnen geschilderten Fällen atrophe Lebersegmente und ahmten teils ein Zirrhose-ähnliches Muster nach. Die Schwierigkeit der präoperativen und histopathologischen Einordnung dieser Läsionen bestand auch hier. Entsprechende Herdbefunde sind in der aktuell gültigen WHO-Klassifikation [17] nicht benannt. Bei zugrunde liegender Verlegung von Lebervenen sind derartige Läsionen auch bei weiteren Grunderkrankungen zu erwarten, so im Rahmen eines Budd-Chiari-Syndroms auf Grundlage einer Hyperkoagulopathie – von uns beobachtet, bislang nicht publiziert.

Lokale, segmentale oder auch Segment-übergreifende Störungen der Leberdurchblutung können zu ausgedehnten Parenchymuntergängen führen. Bei vollständigem Ausfall der arteriohepatischen und portovenösen Perfusion resultieren Infarktnekrosen. Bei erhaltener arteriohepatischer Restperfusion, entsprechend einer relativen Ischämie, wird das hepatozelluläre Parenchym durch eine DR ersetzt. Diese Gallengangsproliferate sind im Vergleich mit den metabolisch hochaktiven Hepatozyten weniger stoffwechselaktiv und resistenter gegenüber einer verminderten Sauerstoffzufuhr bei gestörter Durchblutung. Abhängig vom Ausmaß der Schädigung und der bedingenden Grunderkrankung kann die duktuläre Reaktion ganze Leberazinuseinheiten, in Zirrhosen ganze Parenchymknoten einnehmen. Sogar Lebersegment-übergreifende tumorartige Ausmaße sind möglich. Sie täuschen klinisch-radiologisch hypoperfundiertes Tumorgewebe vor und sind auch histopathologisch zum Teil schwer von einem CCC abzugrenzen. Dies soll unsere Gegenüberstellung eines cholangiozellulären Karzinoms vom „small duct type“ (Abb. 3b, c) demonstrieren. Wenn ganz ausgedehnte Parenchymbereiche der Minderperfusion unterliegen, z. B. im Rahmen eines Budd-Chiari-Syndroms, können aus residuellen Parenchymbezirken mit erhaltener Perfusion hepatozellulär aufgebaute makroregenerative Parenchymknoten erwachsen. Diese sind vergleichsweise hyperperfundiert und können bildmorphologisch ein metastasiertes HCC imitieren [17].

Die Strukturen der MDR sind im Vergleich zu kleinen Gallengängen unreifer gestaltet. Ihre strangförmigen oder auch anastomosierenden Epithelverbände besitzen meist keine Lichtung. Es zeigt sich eine etwas gesteigerte Kernformvarianz bei feingranulärem Kernchromatin. Die Expression von CD56 weist auf eine stammzellnahe Differenzierung hin. Im Gegensatz zu diesen Ischämie-induzierten, Parenchym-ersetzenden, raumfordernden DR zeigen Gallengangsproliferate bei chronischen Gallengangsstenosen meist eine reifere biliäre Morphologie. Sie sind mit ihrer runden Form mit relativ hoch aufgebautem Epithel und Lichtung zum Teil kaum von ortsständigen terminalen Gallengängen zu unterscheiden. Auch die Expression von CD56 ist hier geringer ausgeprägt. Die Expression von epithelialem Membranantigen (EMA) und dem Glykoprotein CD10 sind als Zeichen einer fortgeschritteneren Differenzierung zu werten. Zu beachten ist, dass auch CCC vom „small duct type“ örtlich CD56 exprimieren können.

In der Abgrenzung gegenüber einem hochdifferenzierten CCC vom „small duct type“ oder „malformation type“ [6] kann die Darstellung der Basalmembran in der Versilberungsreaktion (Gordon, Gomori etc.) hilfreich sein (Abb. 3a); so sind bei CCC häufig argyrophile Faserfragmente in den drüsigen Tumorepithelverband integriert. Die irregulär verzweigten Tumordrüsen bilden nicht selten lanzettförmige Enden aus (Abb. 3c) im Gegensatz zu den fingerförmig abgerundeten reaktiven duktulären Proliferaten (Abb. 3b). Zelluläre Atypien, Apoptosen und die meist deutlich höhere proliferative Aktivität auch in sklerosierten Karzinomanteilen weisen auf einen malignen Befund hin. Im Gegensatz zur MDR, die an ihren Rändern kontinuierliche Übergänge zum benachbarten Leberparenchym erkennen lässt, wird an den Karzinomrändern das invasive Tumorwachstum deutlich, insbesondere bei Infiltration in das periportoseptale Stroma. Auch wenn CCC ehemalige Parenchymtrabekel als Leitschienen zur Ausbreitung nutzen, bleibt bei dem Parenchym-ersetzenden Prozess der MDR die bindegewebige lobuläre oder – in Zirrhosen – die knotige Grundstruktur des Lebergewebes erhalten [3, 16]. Zudem sieht man ein eher fein-fibrilläres Hintergrundstroma, das nicht so faserreich oder gar sklerosiert imponiert wie bei Cholangiokarzinomen. Eine weitere Differenzialdiagnose sind kombinierte hepatozelluläre und cholangiozelluläre Karzinome, bei denen der maligne morphologische Aspekt beider Tumorkomponenten zu beachten ist. Schließlich sind auch Gallengangsadenome oder das seltene Cholangiofibrom als benigne Neoplasien gegenüber der MDR abzugrenzen [19]. Hier kann insbesondere die Darstellung der duktulären Aussprossungen der MDR im Bereich der portoparenchymatösen Grenzzone hilfreich sein und im Falle eines Gallengangsadenoms das Fehlen eines zu lokaler Leberatrophie führenden Gefäßverschlusses oder einer anderweitigen Lebererkrankung.

## Ausblick

Wir möchten das Augenmerk auf die neu beschriebene, massive/massebildende duktuläre Reaktion (MDR) der Leber lenken. Die MDR kann präoperativ wie auch vonseiten der Pathologie insbesondere ein Cholangiokarzinom vortäuschen. Sie ist somit eine seltene, aber relevante Differenzialdiagnose hepatischer Herdbefunde. Ihr liegt eine Minderperfusion durch Obliteration des portovenösen Zustroms und/oder venösen Blutabstroms zugrunde, aus der eine lokale tubulär-drüsige Umwandlung des Leberparenchyms folgt. Beobachtete Vorerkrankungen sind Zirrhosen, ausgedehnte Leberparenchymnekrosen, primäre Lebermalignome und Lebermetastasen. Die sorgfältige interdisziplinäre Aufarbeitung und Beschreibung dieser Fälle zur Erfassung weiterer typischer Lebergrunderkrankungen erscheint notwendig. Zudem bieten sie einen makroskopisch fassbaren Einblick in adaptive Leberumbauprozesse bei vorerkrankungsbedingter eingeschränkter Leberregeneration (Tab. 1).

**Tab. 1:** Makroskopische und mikroskopische Aspekte der MDR und ihrer wichtigsten Differenzialdiagnosen nach Fischer et al. [3] und Joldeshova et al. [16]

	MDR	Cholangiozelluläres Karzinom, „small duct type“	Gallengangsadenom
Auftreten bei Kindern und Jugendlichen	Bislang nicht beschrieben	Sehr selten (eher im Kontext von Syndromen)	Sehr selten
Makroskopie	≥ 1,0 cm	In Zirrhosen meist >> 1,0 cm	Meist < 1,0 cm, selten > 2,0 cm
	In Zirrhosen und Nichtzirrhosen	In Zirrhosen und Nichtzirrhosen	Meist keine Zirrhose
	Oft assoziiert mit Parenchymextinktion oder Lebertumor	Lebersegmentunabhängiges, segmentüberschreitendes Wachstum, Satelliten	Oft subkapsulär, meist solitär
	Bereits makroskopisch sichtbare Gefäßobliteration möglich	Selten grob angioinvasives Wachstum	Keine Assoziation mit Gefäßobliteration
	Weiß-grau, derb, relativ scharf umgrenzt. Keine intraläsionalen Nekrosen	Weiß-grau, derb, relativ scharf umgrenzt. Nekrosen möglich	Weiß-grau, derb, relativ scharf umgrenzt. Keine Nekrosen
Histologie	Duktuläre Formationen mit fingerkuppenförmigen Enden, geringe Zellformvarianz, Mitosen äußerst selten	Duktuläre Formationen mit lanzettförmigen Enden, Geringe Zellformvarianz bis zu stärkeren Atypien, nicht selten Mitosen, Einzelzellnekrosen, Apoptosen	Duktuläre Formationen mit fingerkuppenförmigen Enden. Geringe Zellformvarianz, Mitosen äußerst selten
	Intakte Basalmembran	Basalmembranresiduen, Faserfragmente in der Epitheltapete	Intakte Basalmembran
	Fein-fibrilläres Stroma mit diskreter Entzündungszellinfiltration	Lockeres bis sklerosiertes Stroma	Fein-fibrilläres Stroma mit diskreter Entzündungszellinfiltration
	Kein invasives Wachstum	Invasion in portoseptales Stroma	Kein invasives Wachstum
Immunhistochemie	CK7+/CK19+	CK7+/CK19+	CK7+/CK19+
	CD56+ (100 %) [3]	Fokal CD56+ (70–80 %)	CD56 meist +
	Ki-67 < 3 % [3] bzw. < 5 % [16]	Ki-67 </> 10 %	Ki-67: < 5 %
	P53: wt	p53: mut/wt	p53: wt
Molekularpathologische Alterationen	Bislang kein Nachweis charakteristischer Mutationen	*IDH* (20–30 %) [20–22], *FGFR2* (10–20 %) [23–26], *ERBB2* (8 %) [27, 28], *BAP1* (~17 %) [29], *KRAS* (< 10 %) [23], *BRAF* (< 5 %) [30], MSI (2–5 %) [31]	Abhängig vom Kollektiv: *BRAF*-Mutationen in 50 % der Fälle nach Pujals et al. [32, 33], keine *BRAF*-Mutationen nach Sasaki et al. [34]

## Fazit für die Praxis

- Die massive/massebildende duktuläre Reaktion (MDR) (≥ 1,0 cm) stellt einen extensiv-raumfordernden adaptiven Leberumbauprozess auf dem Boden einer relativen Ischämie durch Gefäßverschlüsse im Kontext benigner und maligner Lebererkrankungen dar.
- Die MDR ist bildgebend-präinterventionell, chirurgisch-intraoperativ und histopathologisch-postoperativ als wichtige Differenzialdiagnose insbesondere zu einem Cholangiokarzinom vom „small duct type“ oder einem Gallengangsadenom zu berücksichtigen.

## Data Availability

Alle dieser Arbeit zugrunde liegenden Daten sind in diesem Artikel enthalten.

## Abkürzungen

ALT: Alanin-Amino-Transferase
AST: Aspartat-Amino-Transferase
CCC: Cholangiozelluläres Karzinom
CT: Computertomographie; Computertomogramm
D‑PAS: Diastase-Perjod-Schiff-Säure
EHE: Epitheloides Hämangioendotheliom
EvG: Elastica van Gieson
GGT: Gamma-Glutamyl-Transferase
GOT: Glutamat-Oxalacetat-Transaminase
GPT: Glutamat-Pyruvat-Transaminase
HCC: Hepatozelluläres Karzinom
HE: Hämatoxylin-Eosin
MDR: Massive/mass-forming ductular reaction; massive/massebildende duktuläre Reaktion
MRT: Magnetresonanztomographie
MSI: Mikrosatelliteninstabilität
NCAM: Neural cell adhesion molecule
PAS: Perjod-Schiff-Säure
PBC: Primär biliäre Cholangitis
PSC: Primär sklerosierende Cholangitis
R: Referenzwert, obere Normwertgrenze
